# Global research trends and emerging themes of ketamine in perioperative neurocognitive disorders: a bibliometric analysis

**DOI:** 10.3389/fmed.2026.1960353

**Published:** 2026-10-05

**Authors:** Jun Kang, Ziwei Wang, Jiaxuan Bi, Yongli Duo, Wenjun Yan

**Affiliations:** 1 The First Clinical Medical College of Lanzhou University, Lanzhou, China; 2 Department of Anesthesiology, Gansu Provincial Hospital, Lanzhou, China; 3 The First Clinical Medical College of Gansu University of Chinese Medicine, Lanzhou, China

**Keywords:** bibliometrics, citespace, esketamine, ketamine, perioperative neurocognitive disorders, postoperative cognitive dysfunction, postoperative delirium, VOSviewer

## Abstract

**Background:**

Within the contemporary perioperative neurocognitive disorder (PND) framework, postoperative neurocognitive outcomes include postoperative delirium, delayed neurocognitive recovery, and postoperative neurocognitive disorder. Interest in ketamine and its enantiomers has increased, but the clinical literature on perioperative neurocognitive outcomes remains heterogeneous. This study mapped the global research landscape, collaboration structure, thematic evolution, and recently intensified research themes at the intersection of ketamine and PNDs.

**Methods:**

Publications indexed in the Web of Science Core Collection (WoSCC) and Scopus were retrieved using a prespecified search strategy. English-language articles and reviews published from 2006 to 2025 were included. Records were integrated and deduplicated using a Python-based bibliographic consolidation workflow. VOSviewer 1.6.20 was used for collaboration, citation, and author-keyword analyses, whereas CiteSpace 6.4.R2 Advanced was used for keyword burst detection. A WoSCC-only sensitivity analysis assessed robustness to database composition.

**Results:**

After database restrictions, 559 records remained (WoSCC, *n* = 191; Scopus, *n* = 368); removal of 127 cross-database duplicates yielded 432 unique publications with 13,085 citations. Of these, 283 publications (65.5%) were published during 2021–2025. China contributed the largest number of publications (*n* = 152), whereas the United States had the highest citation count (6,255) and country-level total link strength (66). The most frequent author keywords were delirium (77), postoperative delirium (74), ketamine (65), esketamine (57), and postoperative cognitive dysfunction (55). “Intraoperative ketamine” showed the strongest keyword burst (strength = 4.05; 2021–2023), while recent bursts extending through 2025 included mechanisms, dysfunction, ketamine, esketamine, reliability, and delayed neurocognitive recovery. The WoSCC-only sensitivity analysis showed broadly similar patterns, including a 9/10 overlap among the top keywords (Spearman's *ρ* = 0.784).

**Conclusions:**

Research on ketamine and perioperative neurocognitive outcomes has expanded rapidly, particularly since 2020. Recent literature increasingly emphasizes esketamine, contemporary neurocognitive terminology, mechanistic questions, and delayed neurocognitive recovery. These bibliometric patterns represent research attention rather than evidence of clinical efficacy. Future studies should distinguish ketamine formulations and enantiomers, use standardized PND definitions and outcome windows, and prioritize adequately powered multicenter designs.

## 1 Introduction

Perioperative neurocognitive disorders (PNDs) comprise a spectrum of cognitive disorders identified before and after surgery, including pre-existing neurocognitive disorder, postoperative delirium (POD), delayed neurocognitive recovery within 30 days after surgery, and postoperative neurocognitive disorder from 30 days to 12 months after surgery (1). These conditions differ in timing and diagnostic criteria but collectively reflect clinically relevant disturbances in perioperative cognitive health. More recent clinical guidance, perioperative brain-health consensus recommendations, and European guidelines have reinforced the importance of standardized terminology, preoperative cognitive-risk identification, multidomain prevention, and coordinated perioperative brain-health strategies (2–6).

Proposed mechanisms implicated in PND include neuroinflammation, impaired synaptic plasticity, altered neurotrophic signaling, oxidative stress, and mitochondrial dysfunction (7, 8). Reported rates of postoperative neurocognitive complications vary substantially according to surgical population, outcome definition, and assessment time point, particularly in older adults (9). Postoperative neurocognitive complications are associated with prolonged hospitalization, increased healthcare costs, reduced quality of life, and poorer long-term outcomes (10). Consequently, the clinical diagnosis, treatment, and prevention of PND have emerged as critical areas of research in medicine.

Ketamine is a noncompetitive N-methyl-D-aspartate receptor antagonist with anesthetic, analgesic, and psychotropic properties (11). Beyond its established perioperative roles, interest has grown in whether ketamine or its enantiomers might influence postoperative cognition through anesthetic-sparing effects, modulation of nociception, inflammatory signaling, synaptic plasticity, or other neurobiological pathways (12–14). Clinical findings, however, have been inconsistent. The large PODCAST randomized trial did not demonstrate prevention of postoperative delirium with a single intraoperative subanesthetic ketamine dose in older adults (15). More recently, the POCK multicenter trial similarly found that a single preoperative ketamine bolus did not reduce delayed neurocognitive recovery or postoperative neurocognitive disorder after major orthopedic surgery (16). A 2025 scoping review of 58 clinical studies also emphasized substantial heterogeneity, with most included studies reporting no cognitive benefit (17). By contrast, recent meta-analyses of esketamine trials have suggested possible reductions in short-term POD or delayed neurocognitive recovery, while evidence for longer-term postoperative neurocognitive disorder remains limited (18, 19). Thus, the clinical question remains unresolved and appears sensitive to drug formulation, dose, timing, surgical population, outcome definition, and follow-up interval. A structured overview of the existing literature on ketamine and PND is therefore important for clarifying the research landscape and informing future research priorities.

Bibliometric analysis can complement conventional reviews by quantifying publication growth, identifying productive countries, institutions, authors, and journals, and visualizing collaboration and thematic evolution. Previous bibliometric studies have mapped the broader PND field (20), the global ketamine literature (21), and highly cited POD research (22). However, these broad analyses do not specifically characterize the intersection between ketamine-related perioperative research and postoperative neurocognitive outcomes. This intersection is methodologically important because it spans older POCD terminology, contemporary PND nomenclature, POD research, mechanistic studies, and increasingly differentiated ketamine enantiomers.

Therefore, the present study aimed to provide a focused bibliometric analysis of ketamine in PND-related research from 2006 to 2025. We sought to (1) characterize publication and citation trends; (2) identify major countries, institutions, authors, journals, and highly cited publications; (3) map author-keyword co-occurrence and temporal evolution; (4) identify temporal keyword bursts using CiteSpace; and (5) evaluate the robustness of the merged WoSCC-Scopus results using a WoSCC-only sensitivity analysis. Importantly, the analysis was designed to describe the structure and evolution of the literature rather than to infer clinical efficacy or causal effects.

## 2 Materials and methods

### 2.1 Data sources and search strategy

Bibliographic records were retrieved from the Web of Science Core Collection (WoSCC) and Scopus on 12 September 2026. A 20-year publication window (2006–2025) was prespecified to characterize contemporary development of the field. The analysis was limited to English-language articles and reviews.

The WoSCC Topic search combined a ketamine block (“ketamine”, “esketamine”, S-ketamine variants, R-ketamine variants, “arketamine”, or “racemic ketamine”) with a neurocognitive outcome block containing “perioperative neurocognitive disorder*”, “postoperative neurocognitive disorder*”, “delayed neurocognitive recovery”, “postoperative cognitive dysfunction”, “perioperative cognitive dysfunction”, “postoperative cognitive impairment”, “postoperative cognitive decline”, and “postoperative delirium”, including selected hyphenated variants. The equivalent concept blocks were applied in Scopus using TITLE-ABS-KEY. Complete database-specific search strings are provided in Supplementary Table S1.

### 2.2 Record integration, deduplication, and data harmonization

The initial searches identified 265 records in WoSCC and 525 records in Scopus. After applying the prespecified publication-year, language, and document-type restrictions, 191 WoSCC records and 368 Scopus records remained. Records were integrated with a Python-based Bibliometric-Data-Consolidation-Tool workflow using bibliographic identifiers and normalized metadata to detect cross-database duplicates. A total of 127 duplicate records were merged, producing a final dataset of 432 unique publications.

### 2.3 Descriptive bibliometric indicators

Annual publication counts, cumulative publication output, document types, and total citation counts were calculated from the integrated dataset. Citation counts reflect database values available at the retrieval date and are therefore time-dependent. Country, institution, author, journal, keyword, and document-level rankings were generated from the cleaned bibliographic data. Publication counts were used as the primary ranking metric for countries, institutions, authors, and journals, whereas occurrence frequency was used for keywords. When counts were tied, total link strength was used as the secondary ranking criterion; citation count was used as an additional tie-breaker when necessary. Microsoft Excel 2016 was used to generate the annual and cumulative publication trend visualization (Figure 2).

### 2.4 VOSviewer network and keyword analyses

VOSviewer version 1.6.20 (23) was used for structural bibliometric mapping. Full counting was applied, and association-strength normalization was used for network visualization. Institutional co-authorship analysis required at least five publications, yielding 36 institutions. Author co-authorship analysis required at least 3 publications per author, yielding 50 authors in the final network, which comprised six clusters, 118 links, and a total link strength of 195. Journal-level productivity and connectivity were summarized using publication counts, citations, and total link strength (TLS). R version 4.5.3 was used to generate the journal bubble plot (Figure 3). Citation analysis at the document level was used to identify the 10 most cited publications in the included dataset.

Keyword co-occurrence analysis was based on author keywords. A conservative thesaurus was applied to harmonize obvious variants in capitalization, spacing, hyphenation, spelling, singular/plural form, abbreviations, and clearly equivalent terminology. Conceptually distinct terms were deliberately retained; for example, delirium, postoperative delirium, and emergence delirium were not merged, and ketamine, esketamine, and arketamine were analyzed separately. The final threshold was at least five occurrences, resulting in 60 keywords, seven clusters, 499 links, and a total link strength of 1,053. The same network was used for overlay visualization, in which node color represented average publication year. Detailed normalization rules are listed in Supplementary Table S3.

### 2.5 Citespace burst analysis

CiteSpace version 6.4.R2 Advanced (24) was used specifically for temporal burst detection rather than structural clustering. The time frame was 2006–2025 with one year per slice; the node type was Keyword; term sources included Title, Abstract, Author Keywords (DE), and Keywords Plus (ID); and the g-index was set to *k* = 25. The main manuscript displays the top 29 burst terms. VOSviewer and CiteSpace outputs were treated as complementary rather than directly comparable because the programs use different network construction, normalization, node-selection, and temporal-processing procedures. Software settings and thresholds are summarized in Supplementary Table S2.

### 2.6 Sensitivity analysis

To assess robustness to database composition, a sensitivity analysis compared the full merged dataset (*n* = 432) with the WoSCC-only subset (*n* = 191). For countries, institutions, authors, journals, and keywords, fixed top-10 overlap and Spearman rank correlations were calculated across entities occurring at least twice in the WoSCC-only subset. Ties at the ranking cutoff were not used to modify the fixed top-10 overlap. This analysis was intended to assess the stability of the main bibliometric patterns and did not constitute independent external validation (Supplementary Table S4).

## 3 Results

### 3.1 Literature retrieval and overall characteristics

After application of the prespecified database restrictions, 191 WoSCC records and 368 Scopus records remained. After removal of 127 cross-database duplicates, 432 unique publications were included in the final bibliometric dataset (Figure 1). All 432 publications were in English and were published between 2006 and 2025. The corpus comprised 292 articles (67.6%) and 140 reviews (32.4%). The included publications had accumulated 13,085 citations by the retrieval date.

**Figure 1:**
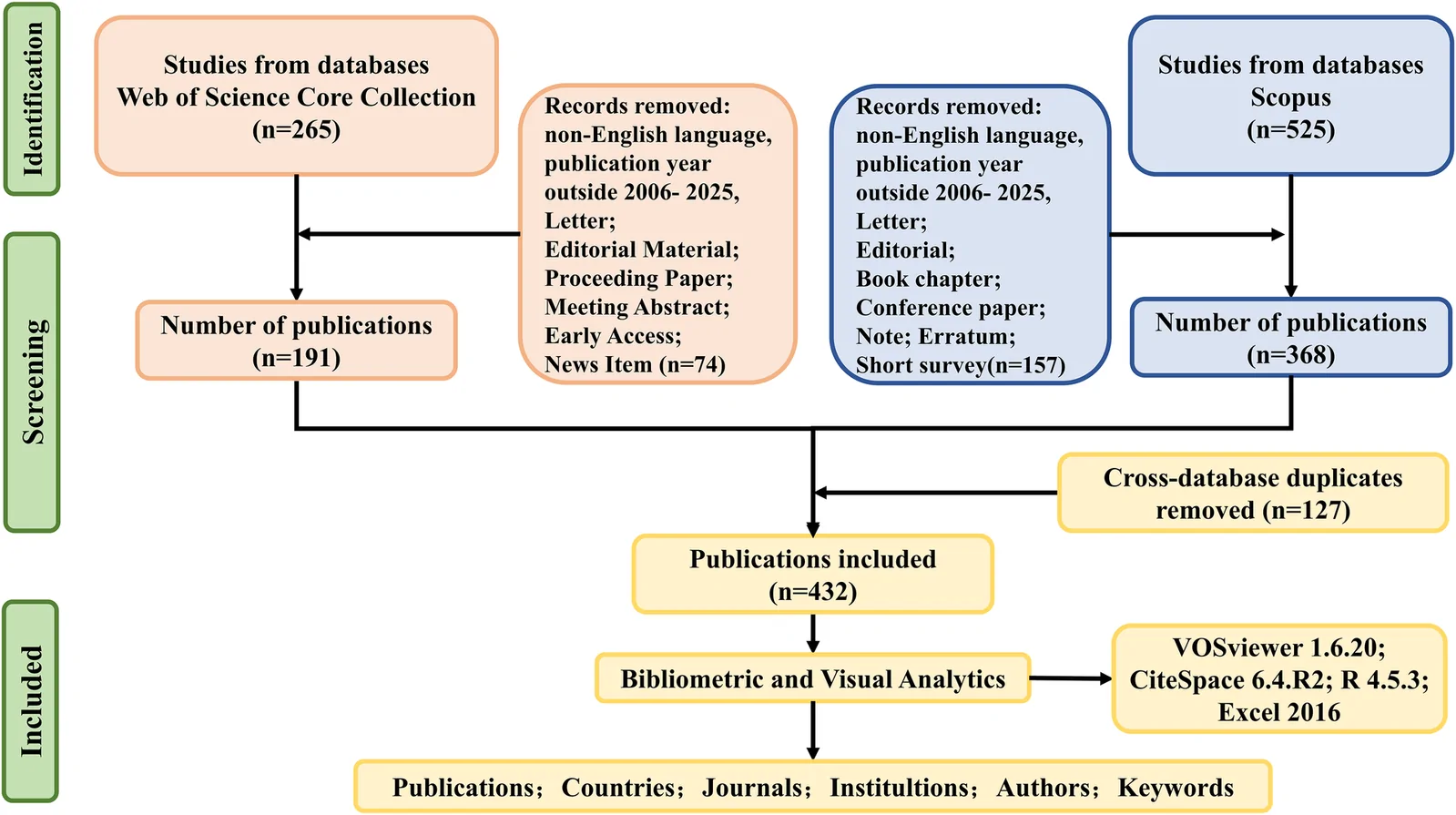
Literature retrieval, database filtering, and data-integration workflow. Records were initially identified from the Web of Science Core Collection (WoSCC; *n* = 265) and Scopus (*n* = 525). After applying the prespecified publication-year, language, and document-type restrictions, 191 WoSCC records and 368 Scopus records remained. The two datasets were subsequently integrated, and 127 cross-database duplicate records were removed, yielding 432 unique publications for the final bibliometric analysis.

### 3.2 Annual publication trends

Annual publication output showed an overall upward trajectory from 2006 to 2025, with a marked acceleration after 2020 (Figure 2). Output increased from four publications in 2006 to 43 in 2021, 49 in 2023, 62 in 2024, and 88 in 2025. In total, 283 publications were published during 2021–2025, accounting for 65.5% of the entire 20-year corpus. These data indicate that most of the literature at the intersection of ketamine and perioperative neurocognitive outcomes is relatively recent.

**Figure 2:**
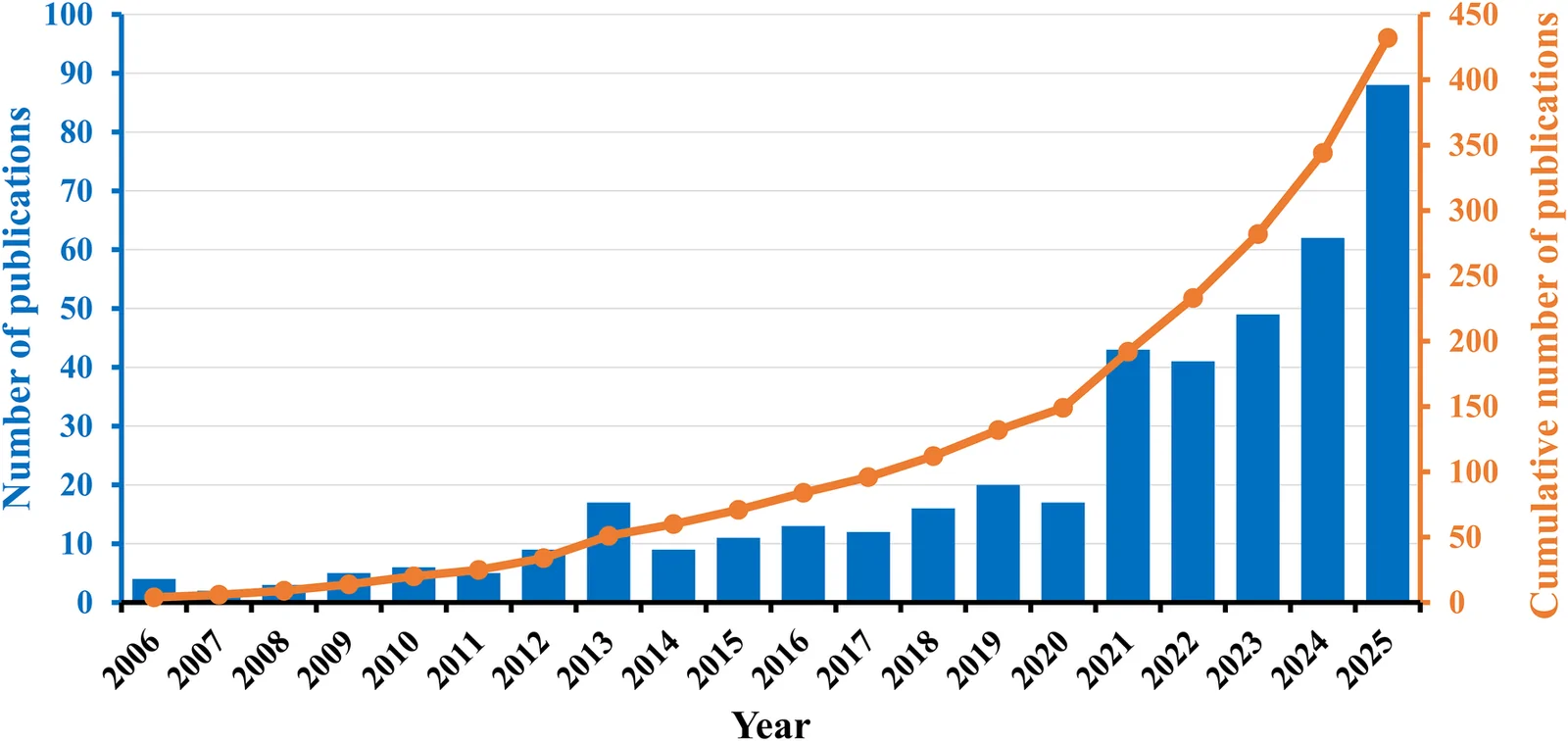
Annual and cumulative publication output from 2006 to 2025. Blue bars represent the annual number of publications, and the orange line represents cumulative publication output. Annual publication activity showed an overall increasing trend, with a marked acceleration after 2020.

### 3.3 Journals and sources

The literature was distributed across a broad range of anesthesia, perioperative, and multidisciplinary journals (Figure 3; Table 1). BMC Anesthesiology was the most productive source with 17 publications, followed by Anesthesia and Analgesia (16) and the British Journal of Anaesthesia (15). The British Journal of Anaesthesia received the highest number of citations among the top 10 productive journals (1,653), followed by Current Opinion in Anesthesiology (519 citations among 10 publications) and the Journal of Cardiothoracic and Vascular Anesthesia (462 citations among 11 publications). The divergence between publication volume and citation rank illustrates that source productivity and citation visibility capture different dimensions of the literature.

**Figure 3:**
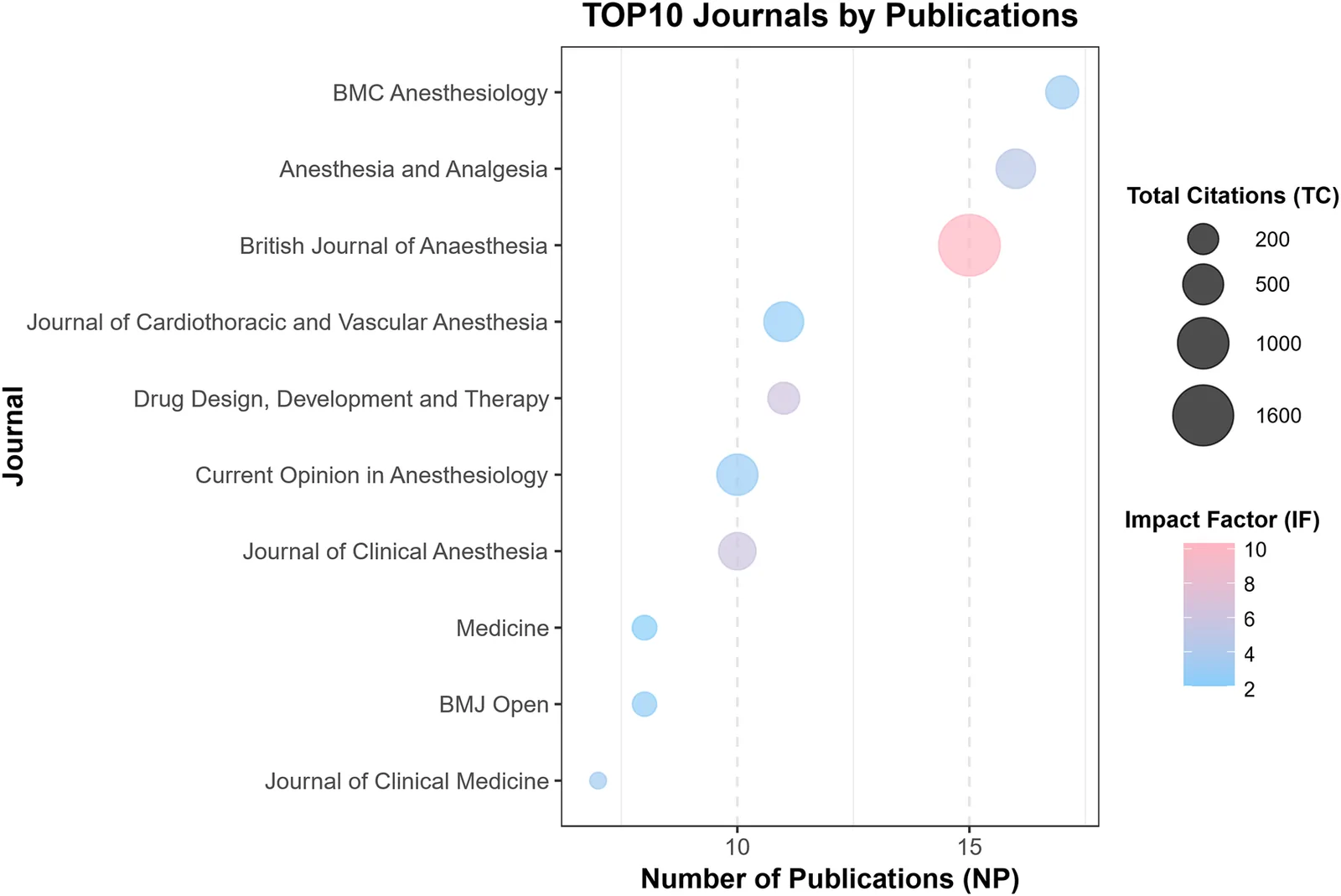
Top 10 journals by publication output. The horizontal position of each bubble represents the number of publications contributed by the journal. Bubble size represents the total citations received by publications from that journal within the analyzed dataset, and bubble color represents the 2025 Journal Impact Factor (JIF).

**Table 1:** Top 10 journals ranked by number of publications.

Rank	Journal	Documents	Citations	Total link strength	2025 journal impact factor
1	*BMC Anesthesiology*	17	251	40	3.2
2	*Anesthesia and Analgesia*	16	452	29	4.7
3	*British Journal of Anaesthesia*	15	1,653	44	10.3
4	*Drug Design, Development and Therapy*	11	221	42	6.1
5	*Journal of Cardiothoracic and Vascular Anesthesia*	11	462	27	2.6
6	*Journal of Clinical Anesthesia*	10	380	19	6.0
7	*Current Opinion in Anesthesiology*	10	519	5	2.8
8	*BMJ Open*	8	82	27	2.5
9	*Medicine*	8	87	7	2.0
10	*Journal of Clinical Medicine*	7	36	11	3.3

TLS, total link strength; JIF, Journal Impact Factor.

### 3.4 Countries and international collaboration

China ranked first in publication output with 152 documents, followed closely by the United States with 145 (Table 2; Figure 4). Although the United States published slightly fewer papers, it had the highest citation count (6,255) and the greatest country-level TLS (66). China accumulated 2,913 citations and a TLS of 28. The United Kingdom had a TLS of 30, followed by China (28) and Canada (27), indicating substantial international connectivity among these countries. Canada, the United Kingdom, Italy, Germany, Switzerland, France, India, and Australia also ranked among the 10 most productive countries.

**Table 2:** Top 10 countries contributing to ketamine-related PND research.

Rank	Country	Documents	Citations	Total link strength
1	China	152	2,913	28
2	United States	145	6,255	66
3	Canada	21	1,033	27
4	United Kingdom	19	1,177	30
5	Italy	16	1,145	20
6	Germany	15	829	14
7	Switzerland	14	951	17
8	France	13	221	7
9	India	12	765	11
10	Australia	11	1,321	21

**Figure 4:**
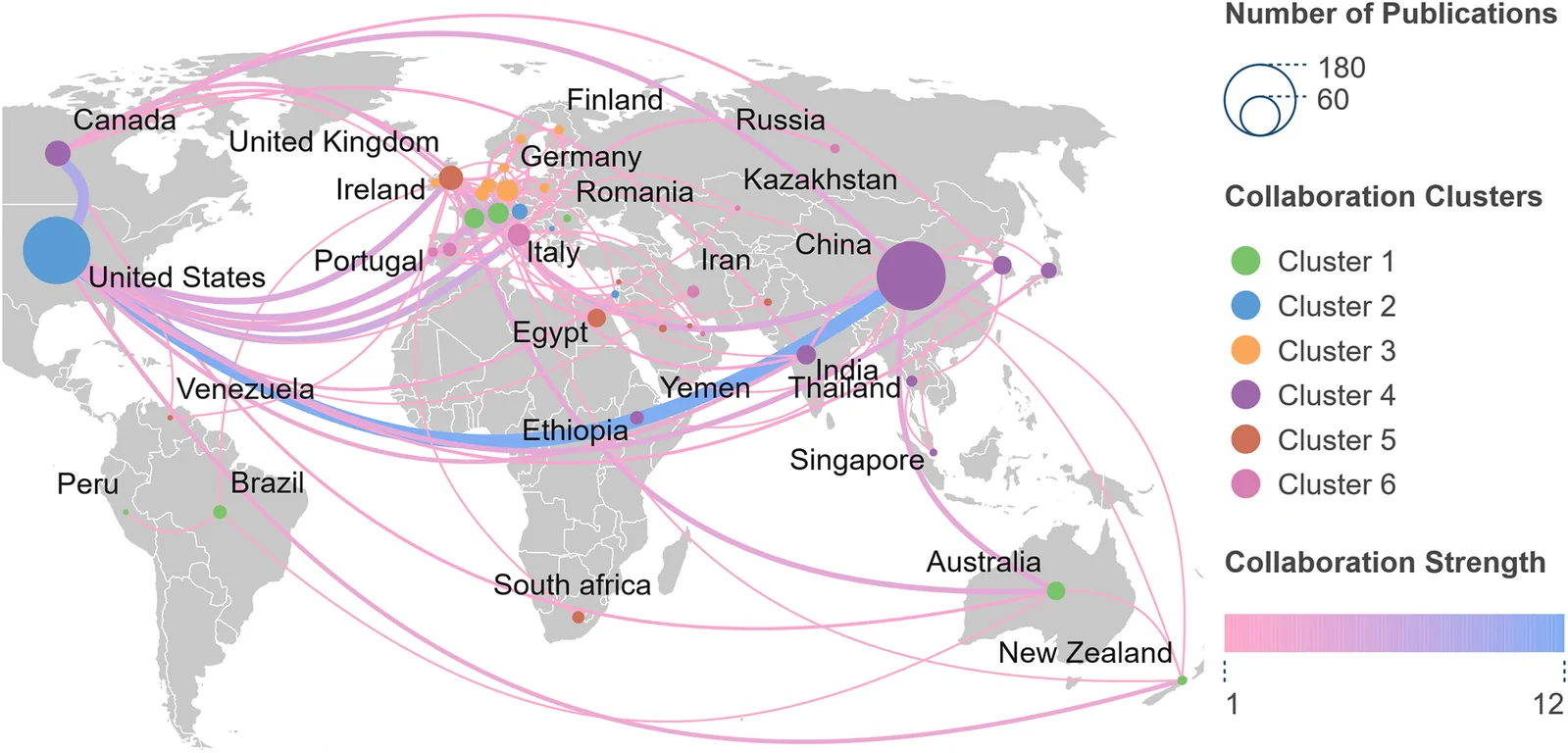
Global distribution and international collaboration network of countries. Node size represents publication output, and node color indicates collaboration-cluster membership. Curved links represent international co-authorship relationships; link width and color intensity reflect the strength of bilateral collaboration between country pairs.

### 3.5 Institutional collaboration

Among institutions meeting the network threshold, Weill Cornell Medicine was the most productive institution with 15 publications, followed by Zhengzhou University with 14 (Table 3; Figure 5). Harvard University, Massachusetts General Hospital, Capital Medical University, and Huazhong University of Science and Technology each contributed 11 publications. Weill Cornell Medicine also had the highest citation count (1,858) and institutional TLS (29), followed by Harvard University (1,049 citations; TLS = 23) and Massachusetts General Hospital (493 citations; TLS = 17). These findings indicate a geographically distributed institutional network involving major centers in the United States and China.

**Table 3:** Top 10 institutions contributing to ketamine-related PND research.

Rank	Institution	Documents	Citations	Total link strength
1	Weill Cornell Medicine	15	1,858	29
2	Zhengzhou University	14	254	6
3	Harvard University	11	1,049	23
4	Massachusetts General Hospital	11	493	17
5	Capital Medical University	11	441	4
6	Huazhong University of Science and Technology	11	265	4
7	Tianjin Medical University	8	138	4
8	Sichuan University	8	96	0
9	Washington University in St. Louis	7	648	12
10	Icahn School of Medicine at Mount Sinai	7	494	11

**Figure 5:**
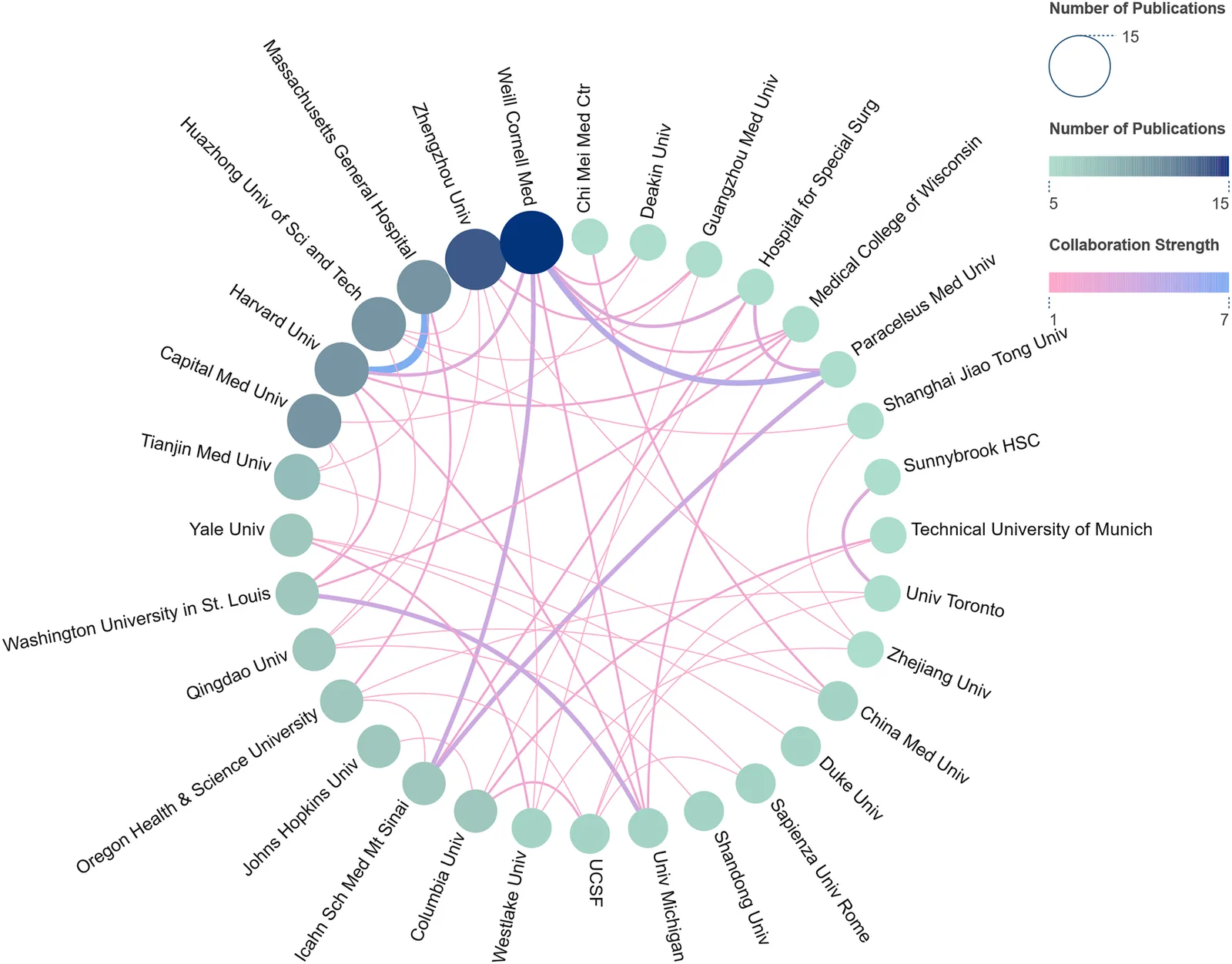
Institutional collaboration network in ketamine-related perioperative neurocognitive research. Institutions with at least five publications were included. Node size and node color intensity represent publication output, whereas the width and color intensity of connecting lines indicate collaboration strength between institutions. Larger and darker nodes therefore represent institutions with greater publication output.

### 3.6 Author productivity and collaboration

Zhang Yiying was the most productive author with 12 publications and 336 citations, followed by Yang Jianjun with eight publications (Table 4; Figure 6). Liu Xin and Li Jiaqi each contributed seven publications, while Zhou Yang and Federico Bilotta each contributed six. Among the top 10 authors, Crispiana Cozowicz, Stavros G. Memtsoudis, and Jashvant Poeran had the highest TLS (19 each), reflecting comparatively strong co-authorship connectivity within the mapped network. George A. Mashour had the highest citation count among the top 10 productive authors (540 citations).

**Table 4:** Top 10 authors contributing to ketamine-related PND research.

Rank	Author	Documents	Citations	Total link strength
1	Zhang, Yiying	12	336	11
2	Yang, Jianjun	8	37	3
3	Liu, Xin	7	67	4
4	Li, Jiaqi	7	185	3
5	Zhou, Yang	6	58	3
6	Bilotta, Federico	6	462	0
7	Cozowicz, Crispiana	5	308	19
8	Memtsoudis, Stavros G.	5	308	19
9	Poeran, Jashvant	5	308	19
10	Mashour, George A.	5	540	7

**Figure 6:**
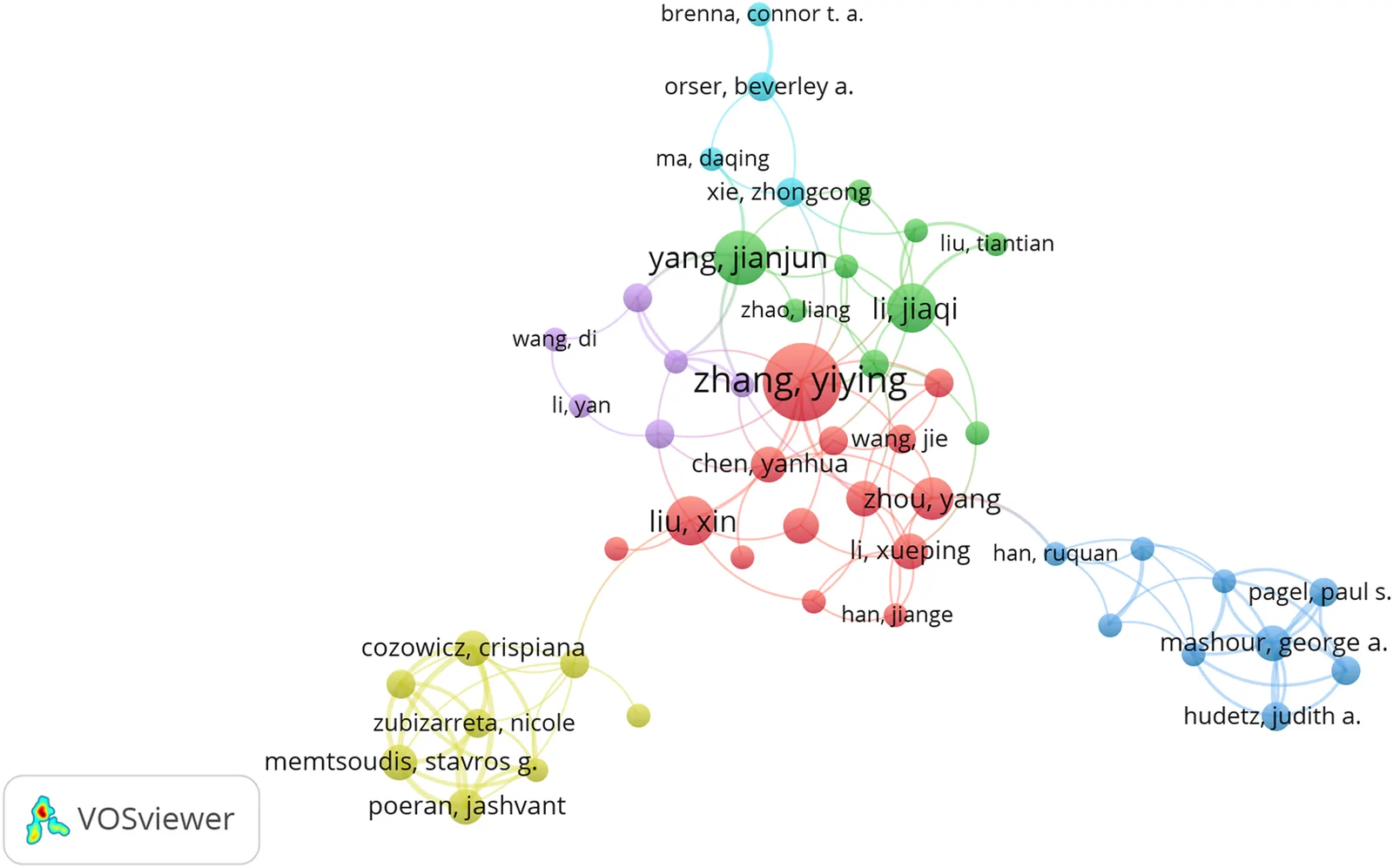
Author co-authorship network in ketamine-related perioperative neurocognitive research. The final co-authorship network contained 50 authors. Node size represents publication output, node color denotes co-authorship cluster membership, and link thickness reflects the strength of collaboration between authors.

### 3.7 Highly cited publications

The 10 most cited publications in the included dataset are presented in Table 5. The most cited article was the 2017 international multicenter PODCAST randomized trial by Avidan et al., with 434 citations (15). This was followed by the 2021 randomized trial of anaesthetic depth and postoperative delirium by Evered et al. (345 citations) (25) and the 2024 updated European guideline on postoperative delirium by Aldecoa et al. (305 citations) (3). Other highly cited publications addressed delirium pathophysiology, neuroinflammation, POCD mechanisms, cardiac surgery, hip-fracture delirium, anesthetic neurotoxicity, and delirium prevention. Accordingly, the highly cited publications spanned multiple clinical and mechanistic domains rather than focusing solely on ketamine efficacy.

**Table 5:** Top 10 most cited publications in the included dataset.

Rank	First author	Year	Title	Journal	Citations
1	Avidan MS	2017	Intraoperative ketamine for prevention of postoperative delirium or pain after major surgery in older adults: an international, multicentre, double-blind, randomised clinical trial	*The Lancet*	434
2	Evered L	2021	Anaesthetic depth and delirium after major surgery: a randomised clinical trial	*British Journal of Anaesthesia*	345
3	Aldecoa C	2024	Update of the European Society of Anaesthesiology and Intensive Care Medicine evidence-based and consensus-based guideline on postoperative delirium in adult patients	*European Journal of Anaesthesiology*	305
4	Maldonado J	2017	Acute Brain Failure: Pathophysiology, Diagnosis, Management, and Sequelae of Delirium	*Critical Care Clinics*	301
5	Safavynia S	2019	The role of neuroinflammation in postoperative cognitive dysfunction: Moving from hypothesis to treatment	*Frontiers in Psychiatry*	290
6	Skvarc DR	2018	Post-Operative Cognitive Dysfunction: An exploration of the inflammatory hypothesis and novel therapies	*Neuroscience & Biobehavioral Reviews*	275
7	Hudetz JA	2009	Ketamine Attenuates Delirium After Cardiac Surgery With Cardiopulmonary Bypass	*Journal of Cardiothoracic and Vascular Anesthesia*	253
8	Juliebø V	2009	Risk factors for preoperative and postoperative delirium in elderly patients with hip fracture	*Journal of the American Geriatrics Society*	236
9	Jevtovic-Todorovic V	2013	Anaesthetic neurotoxicity and neuroplasticity: an expert group report and statement based on the BJA Salzburg Seminar	*British Journal of Anaesthesia*	218
10	Janssen T	2019	Prevention of postoperative delirium in elderly patients planned for elective surgery: Systematic review and meta-analysis	*Clinical Interventions in Aging*	203

### 3.8 Author-keyword co-occurrence and thematic structure

After thesaurus-based standardization, 60 author keywords met the minimum threshold of five occurrences and formed seven clusters in the VOSviewer network (Figure 7A). The most frequent keyword was “delirium” (77 occurrences; TLS = 182), followed by “postoperative delirium” (74; TLS = 148), “ketamine” (65; TLS = 129), “esketamine” (57; TLS = 113), and “postoperative cognitive dysfunction” (55; TLS = 103) (Table 6). Other prominent terms included “anesthesia” (50; TLS = 109), “older adults” (46; TLS = 103), “dexmedetomidine” (32; TLS = 88), “cognitive dysfunction” (22; TLS = 58), and “cardiac surgery” (22; TLS = 45). The dense connections among delirium, POD, ketamine, esketamine, POCD, anesthesia, and older-adult terms reflect the clinical and conceptual overlap represented in this literature.

**Figure 7:**
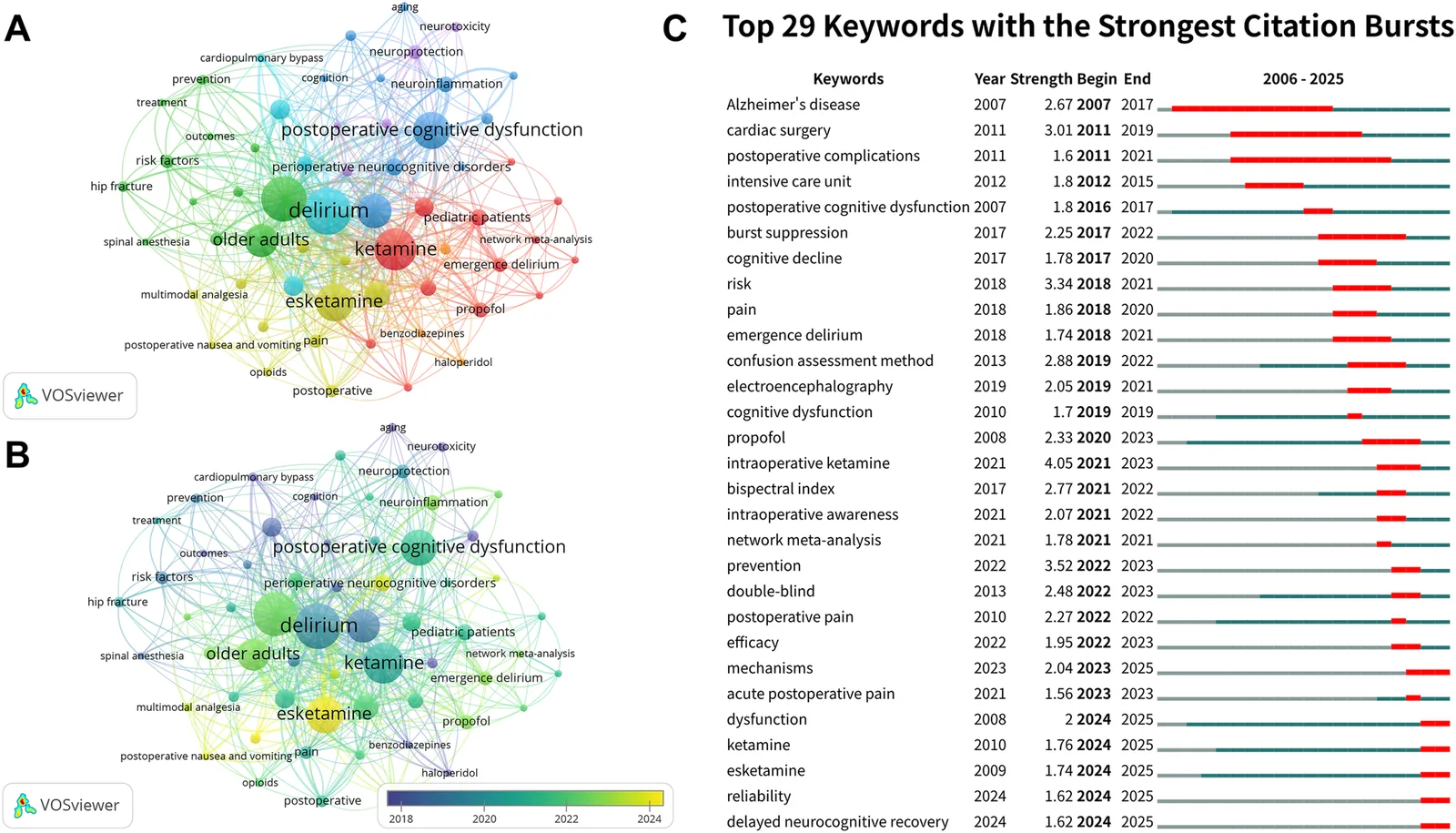
Keyword co-occurrence, temporal overlay, and burst analyses. **(A)** Author-keyword co-occurrence network. Node size represents keyword occurrence frequency, node color indicates cluster membership, and link thickness reflects co-occurrence strength. **(B)** Overlay visualization of the same author-keyword network. Node color represents the average publication year (APY), with colors toward the later end of the scale indicating more recent average use. **(C)** Keyword burst analysis generated with CiteSpace. The 29 detected burst terms are arranged according to the beginning year of each burst. Red segments indicate periods of intensified keyword occurrence.

**Table 6:** Top 10 keywords ranked by occurrence frequency.

Rank	Keyword	Occurrences	Total link strength
1	Delirium	77	182
2	Postoperative delirium	74	148
3	Ketamine	65	129
4	Esketamine	57	113
5	Postoperative cognitive dysfunction	55	103
6	Anesthesia	50	109
7	Older adults	46	103
8	Dexmedetomidine	32	88
9	Cognitive dysfunction	22	58
10	Cardiac surgery	22	45

### 3.9 Temporal evolution and keyword bursts

The VOSviewer overlay visualization (Figure 7B) suggested a temporal shift from older terminology and established perioperative contexts toward more recent emphasis on esketamine, older adults, contemporary PND terminology, and delirium-focused research. Keyword burst analysis further characterized the temporal evolution of research attention (Figure 7C). “Intraoperative ketamine” exhibited the strongest burst (strength = 4.05; 2021–2023), followed by “prevention” (3.52; 2022–2023), “risk” (3.34; 2018–2021), “cardiac surgery” (3.01; 2011–2019), and “confusion assessment method” (2.88; 2019–2022). Bursts involving “bispectral index” (2.77; 2021–2022), “propofol” (2.33; 2020–2023), and “burst suppression” (2.25; 2017–2022) reflected sustained attention to anesthetic management and perioperative brain monitoring. Terms whose bursts extended through 2025 included “mechanisms” (2.04; 2023–2025), “dysfunction” (2.00; 2024–2025), “ketamine” (1.76; 2024–2025), “esketamine” (1.74; 2024–2025), “reliability” (1.62; 2024–2025), and “delayed neurocognitive recovery” (1.62; 2024–2025). These bursts were interpreted as recently intensified research attention rather than evidence of established clinical efficacy or validated research frontiers.

### 3.10 Sensitivity analysis

The WoSCC-only sensitivity analysis showed broadly similar bibliometric patterns to those observed in the merged dataset. Spearman rank correlations between the merged corpus and the WoSCC-only subset were 0.802 for countries, 0.824 for institutions, 0.687 for authors, 0.679 for journals, and 0.784 for keywords. Fixed top-10 overlap was 6/10 for countries, 7/10 for institutions, 6/10 for authors, 6/10 for journals, and 9/10 for keywords. Thus, contributor-level rankings were somewhat sensitive to database coverage, whereas the thematic structure was particularly stable. The sensitivity analysis is reported in Supplementary Table S4.

## 4 Discussion

### 4.1 Principal findings

This bibliometric analysis shows that ketamine-related perioperative neurocognitive research has evolved from a relatively limited body of literature into a rapidly expanding interdisciplinary area. More importantly than the increase in publication volume itself, the thematic structure reveals two parallel developments: persistent clinical concern regarding delirium and postoperative cognitive outcomes, and increasing differentiation of drug formulation, neurocognitive terminology, perioperative brain monitoring, and mechanistic research. The coexistence of legacy POCD terminology with contemporary PND concepts, together with the recent prominence of esketamine and delayed neurocognitive recovery, suggests that the field is undergoing conceptual as well as quantitative expansion.

### 4.2 Rapid growth of a converging research field

The marked increase in publications after 2020 likely reflects convergence of several developments. The 2018 PND nomenclature recommendations provided a more coherent temporal framework for perioperative cognitive outcomes (1), and subsequent clinical guidance has emphasized perioperative brain health, delirium prevention, electroencephalographic monitoring, and risk reduction (2, 3). At the same time, ketamine research expanded across anesthesia, analgesia, psychiatry, and neurobiology. A recent bibliometric analysis of the broader ketamine field documented sustained growth and identified perioperative applications as one of several major themes (21), while a broad PND bibliometric study reported increasing attention to prevention, older patients, and dexmedetomidine (20). The present analysis indicates that ketamine-related neurocognitive research represents a rapidly expanding intersection between the broader ketamine and PND literatures.

Importantly, this growth should not be interpreted simply as increasing interest in ketamine as a perioperative drug. It more likely reflects convergence of previously partly separate research traditions. Delirium research, older POCD literature, contemporary PND nomenclature, perioperative brain monitoring, and ketamine pharmacology increasingly address overlapping clinical questions but retain different conceptual and methodological backgrounds. The coexistence of older POCD terminology with newer PND concepts in the present dataset therefore reflects gradual conceptual integration rather than abrupt replacement of one research paradigm by another.

### 4.3 Geographic, institutional, and journal structure

The geographic and institutional patterns highlight an important distinction between research productivity, citation visibility, and collaborative connectivity. High publication output did not necessarily coincide with high citation or network metrics, because these indicators capture different dimensions of scientific activity and are influenced by publication age, database coverage, disciplinary visibility, and collaboration structure. Similar geographic concentration and cross-national differences in bibliometric performance have also been reported in broader analyses of PND and ketamine research (20, 21). These indicators should therefore not be interpreted as direct measures of research quality.

The distribution of leading institutions across multiple centers suggests that the field is supported by several perioperative and neuroscience research communities rather than a single dominant group. Likewise, the presence of both specialist anesthesia journals and broader multidisciplinary outlets reflects the interdisciplinary nature of the topic. From a clinical-research perspective, broader international collaboration may facilitate harmonization of neurocognitive definitions and assessment methods, increase population diversity, and improve the external validity of future studies.

### 4.4 Highly cited literature and the ketamine efficacy question

The most cited publication in the dataset was the PODCAST trial by Avidan et al. (15). This is notable because the trial did not support routine use of a single subanesthetic intraoperative ketamine dose for preventing postoperative delirium in older adults. A later large multicenter study, the POCK trial, similarly found no reduction in delayed neurocognitive recovery at seven days or postoperative neurocognitive disorder at three months after a 0.5 mg/kg preoperative ketamine bolus in older patients undergoing major orthopedic surgery (16). The prominence of these trials is consistent with the strong bursts observed for “intraoperative ketamine” and “prevention”, suggesting sustained research attention to an unresolved clinical question rather than uniform positive evidence.

The recent evidence base remains heterogeneous. Brenna et al. reviewed 58 adult surgical studies and reported cognitive benefit in a minority of studies, whereas most did not demonstrate benefit (17). In contrast, recent meta-analyses of esketamine trials have reported lower risks of short-term POD or delayed neurocognitive recovery (18, 19). However, these pooled estimates were based on relatively small trials, many conducted in Chinese populations, and evidence for neurocognitive outcomes at three months was limited (18). These findings reinforce the need to distinguish racemic ketamine from S-ketamine/esketamine and R-ketamine/arketamine in both bibliometric interpretation and future clinical research. The increasing prominence of “esketamine” in our keyword network should therefore be viewed as a marker of research diversification, not proof that one formulation is clinically superior.

These apparently divergent findings may therefore reflect clinical and methodological heterogeneity rather than a simple conflict between positive and negative evidence. Formulation, dose, timing, route of administration, surgical population, background anesthetic regimen, and neurocognitive assessment window all differ across studies. Consequently, future evidence synthesis should avoid treating “ketamine” as a homogeneous intervention and should examine whether treatment effects vary according to formulation, patient vulnerability, and the specific neurocognitive phenotype being measured.

### 4.5 Delirium, older adults, and the evolution of PND terminology

The coexistence of “delirium”, “postoperative delirium”, “emergence delirium”, POCD, and contemporary PND terminology illustrates a fundamental challenge in this literature: related perioperative neurocognitive outcomes have often been studied under overlapping but non-equivalent labels. This distinction is clinically important rather than merely terminological. POD is an acute fluctuating neuropsychiatric syndrome (26, 27), whereas delayed neurocognitive recovery and postoperative neurocognitive disorder are defined using different time windows and cognitive assessment approaches. Emergence delirium represents yet another peri-anesthetic context and should not be treated as synonymous with POD or later cognitive decline (28).

The simultaneous prominence of “postoperative cognitive dysfunction” and the recent burst of “delayed neurocognitive recovery” also captures an important terminological transition. POCD remains deeply embedded in older studies and continues to appear in contemporary publications, whereas the 2018 framework recommends time-defined PND terminology (1). Retaining these terms separately allows the map to display this historical layering. Future studies would benefit from adopting standardized outcome definitions, clearly separating POD, delayed neurocognitive recovery, and postoperative neurocognitive disorder, and aligning assessment tools with the relevant postoperative interval.

The prominence of older adults is similarly better interpreted through vulnerability than through chronological age alone. Baseline cognitive impairment, frailty, sensory deficits, depression, comorbidity burden, and delirium susceptibility may all modify postoperative neurocognitive outcomes (29, 30). Future studies should therefore characterize baseline vulnerability more comprehensively when defining high-risk populations.

### 4.6 Mechanisms, neuroinflammation, and perioperative brain monitoring

Mechanistic interest was visible in the recent burst of “mechanisms” and in the broader keyword environment containing neuroinflammatory and anesthesia-related terms. Neuroinflammation has long been proposed as one pathway linking surgery, anesthesia, and postoperative cognitive change (31), and ketamine has plausible anti-inflammatory and synaptic effects (32). However, bibliometric prominence does not validate a mechanism, and mechanistic explanations derived from preclinical models cannot be assumed to account for clinical PND. This distinction is particularly important because neurocognitive outcomes are influenced by baseline vulnerability, surgical stress, pain, sleep disruption, hemodynamics, medication exposure, infection, and numerous other perioperative factors.

A multifactorial framework may therefore be more appropriate than a single drug–single mechanism model. Ketamine may theoretically modify several pathways simultaneously through analgesia, anesthetic-sparing effects, inflammatory signaling, or synaptic mechanisms, yet the net neurocognitive effect may differ according to baseline vulnerability, surgical stress, concomitant anesthetic exposure, and the postoperative time point examined. This provides one possible explanation for why mechanistic plausibility and clinical trial outcomes do not always align.

The earlier bursts of “bispectral index”, “burst suppression”, and “propofol” indicate that ketamine-PND research is embedded within a broader perioperative brain-monitoring literature. The highly cited randomized trial by Evered et al. linked lighter anesthetic depth with lower POD incidence in a selected major-surgery population (25), although the generalizability and mechanism of this relationship remain debated. Contemporary PND prevention is therefore better conceptualized as a multidomain problem than as a single-drug question, consistent with current European and clinical guidance (2, 3, 5, 33).

### 4.7 Implications for future research

The present findings suggest three priorities for future research. First, neurocognitive outcomes require greater standardization. Studies should adopt contemporary PND terminology or explicitly map legacy POCD definitions to current categories, distinguish POD from delayed neurocognitive recovery and later postoperative neurocognitive disorder, and use prespecified assessment windows and validated instruments. Greater harmonization is essential if future trials are to be meaningfully compared or pooled.

Second, ketamine exposure should be characterized more precisely. Racemic ketamine, esketamine, and arketamine should not be treated as interchangeable interventions. Dose, route, timing, single vs. repeated administration, background anesthetic technique, analgesic strategy, and surgical context should be reported systematically. Such stratification may help determine whether inconsistent findings reflect biological differences between formulations or heterogeneity in intervention design.

Third, future studies should move toward phenotype-informed, adequately powered multicenter designs. Baseline cognition, frailty, depression, sensory impairment, comorbidity burden, and delirium vulnerability should be characterized prospectively, particularly in older adults. Mechanistic biomarkers or physiological measures should, where possible, be linked to prespecified clinical phenotypes rather than interpreted independently. This approach may help move the field from identifying bibliometric and clinical associations toward testing biologically coherent and clinically meaningful hypotheses.

### 4.8 Limitations

Several limitations should be acknowledged. First, although combining WoSCC and Scopus improves coverage, no bibliographic database is exhaustive, and database indexing practices may influence country, institution, author, journal, and citation rankings. Second, citation counts are time-dependent and favor older publications; high citation frequency should not be equated with methodological quality or clinical importance. Third, author and institutional disambiguation is imperfect in bibliographic metadata despite cleaning, particularly for common names and multi-institution affiliations. Fourth, the search strategy was intentionally focused on explicit perioperative/postoperative neurocognitive terminology. It may miss relevant studies that report cognitive outcomes without using the selected terms. Fifth, terminology across the literature remains heterogeneous. POCD, cognitive dysfunction, cognitive impairment, delayed neurocognitive recovery, and postoperative neurocognitive disorder are not fully interchangeable, and older studies predate contemporary nomenclature. We therefore used conservative normalization and preserved conceptually distinct terms, but residual semantic heterogeneity is unavoidable. The restriction to English-language publications may have introduced language bias. In addition, citation counts were used as reported by the source databases and self-citations were not separately excluded; therefore, citation-based indicators may partly reflect author or research-group citation practices.

## 5 Conclusion

The global literature on ketamine and perioperative neurocognitive outcomes expanded substantially from 2006 to 2025, with particularly rapid growth after 2020. China and the United States were the leading contributors, while major institutions in the United States and China formed important collaboration hubs. Delirium, postoperative delirium, ketamine, esketamine, POCD, anesthesia, and older adults constituted the principal thematic core. Recent bursts involving mechanisms, ketamine, esketamine, and delayed neurocognitive recovery indicate intensified attention to formulation-specific questions and contemporary neurocognitive terminology. Future progress in this field would benefit from standardized PND definitions, clearer differentiation among ketamine formulations and enantiomers, harmonized neurocognitive assessments, and adequately powered multicenter clinical studies across diverse surgical populations.

## Data Availability

Publicly available datasets were analyzed in this study. This data can be found here: The data analyzed in this study were retrieved from two publicly available academic databases: Web of Science Core Collection (https://www.webofscience.com) and Scopus (https://www.scopus.com). The complete retrieval strategy and literature inclusion criteria are described in detail in the Methods section of the manuscript, and the corresponding dataset can be fully reproduced by following the specified search formulas. No dedicated accession number is assigned to this literature-based dataset.
